# Supplementary material for: Nitric Oxide in the Field: Prevalence and Use of Nitrates by Dietitians and Nutritionists in Spanish Elite Soccer
Source: Nutrients. 2023 Dec 16;15(24):5128. doi: 10.3390/nu15245128 (PMC10745965; doi:10.3390/nu15245128)
Supplement: Supplementary file 1 [file nutrients-15-05128-s001.zip › nutrients-2770436-supplementary.pdf]

USE OF NITRATES AS AN ERGOGENIC AID IN ELITE FOOTBALL

Dear colleague, we are conducting a study on nitrate intake in football players. The aim of this study is to assess the use and behaviours related to nitrate intake in Spanish elite football clubs as a necessary first step to provide pragmatic and evidence-based guidance for safe and effective nitrate supplementation. Your collaboration will be of great help to us, and we are very grateful to you for answering these questions.

Your identity will remain anonymous, and the data will only be used statistically in order to know the reality of nitrate consumption in football. The Organic Law on Data Protection will be respected at all times. This study has been approved by the ethics committee of the University of Valencia with registration number 1534145.

CONSENT

- I have been informed of the characteristics of the research project entitled: "Nitric oxide in the field: prevalence and use of nitrates by dietitians-nutritionists in spanish elite soccer".
- I have read the information on the project, and I have been able to formulate the doubts that have arisen in this respect. I consider that I have understood this information.
- I am informed of the possibility to withdraw from the study at any time.
- I am informed about how my data will be processed.

By virtue of these conditions, I consent to participate in this study. Consent is given by the completion of the survey.

Do not fill in more than one entry per person and write in CAPITAL LETTERS all answers that require text.

* Indica que la pregunta es obligatoria

1. Sex: * ***Required*** *

Selecciona todas las opciones que correspondan.

- [ ] Female
- [ ] Male
- [ ] I prefer not to say

2. What is your role in the club? *

Selecciona todas las opciones que correspondan.

- [ ] Nutritionist
- [ ] Dietitian
- [ ] Internship student
- [ ] Other

3. In which division does the team(s) you primarily supervise play? * ***Required*** *

Selecciona todas las opciones que correspondan.

- [ ] 1st Division: LALIGA SANTANDER
- [ ] 2nd Division: LALIGA SMARTBANK
- [ ] 1st RFEF Division
- [ ] 2nd RFEF Division
- [ ] 3rd RFEF División
- [ ] Regional categories
- [ ] 1st RFEF Femenina
- [ ] 2nd RFEF Femenina

4. Does your club provide nitrates to players as a nutritional supplement? * ***Required*** *

Marca solo un óvalo.

- ( ) Yes *Ir a la pregunta 7*
- ( ) No *Ir a la pregunta 5*

If the answer is no:

5. What is the reason for not using nitrate supplementation? * ***Required*** *

Selecciona todas las opciones que correspondan.

- [ ] Lack of knowledge of its use
- [ ] Limitations of the club (financial, sponsors...)
- [ ] Footballer's rejection
- [ ] Own decision
- [ ] Technical/medical staff decision

6. Has the club used nitrate supplementation before? * ***Required***

Selecciona todas las opciones que correspondan.

- [ ] Yes
- [ ] No
- [ ] I don't know

If the answer is yes:

7. How long has the team been using nitrates as a supplement? * ***Required*** *

Selecciona todas las opciones que correspondan.

- [ ] <1 year
- [ ] 1-3 years
- [ ] + 3 years
- [ ] Not sure

8. What is the main reason for using nitrates as a supplement? (You can select more than one option) * ***Required*** *

Selecciona todas las opciones que correspondan.

- [ ] Personal experience with nitrates
- [ ] Team's commercial agreements with the supplementation brand
- [ ] Recommendations from fellow nutritionists
- [ ] Scientific research

9. When do players consume nitrates? (can be both) * ***Required*** *

Selecciona todas las opciones que correspondan.

- [ ] Match
- [ ] Training

10. On match days when are they used? (select more than one if necessary) *
* **_Required_**

Selecciona todas las opciones que correspondan.

- [ ] <1 hour before the match
- [ ] 1-2 hours before the match
- [ ] 2-3 hours before the match
- [ ] >3 hours before the match
- [ ] At half-time of the match
- [ ] At match stoppages
- [ ] >1 hour after the match
- [ ] 1-2 hours after the match
- [ ] 2-3 hours after the match
- [ ] >3 hours after the match

11. When do you supplement in training? (select more than one if necessary) * **_Required_** *

Selecciona todas las opciones que correspondan.

- [ ] Not used
- [ ] Casual
- [ ] <1 hour pre-training
- [ ] 1-2 hours pre-training
- [ ] 2-3 hours pre-training
- [ ] >3 hours pre-training
- [ ] During training
- [ ] <1 hour post-training
- [ ] 1-2 hours post-training
- [ ] 2-3 hours post-training
- [ ] >3 hours post-training

12. In what form are nitrates prescribed to players? (select more than one if necessary) * ***Required*** *

Selecciona todas las opciones que correspondan.

- [ ] Beetroot shots
- [ ] Powdered
- [ ] Beetroot juice
- [ ] Beetroot juice concentrate
- [ ] Gels
- [ ] Capsules
- [ ] Porridge
- [ ] Pre-workout
- [ ] Otros: ____________________

13. Please specify the brand of supplementation (more than one can be used) * ***Required*** *

____

14. What dose of nitrates is provided? (indicate unknown if needed) * ***Required*** *

____

15. Are the doses of nitrates supplied the same each time they are used? * ***Required*** *

Marca solo un óvalo.

- ( ) Yes
- ( ) No

16. Do all players follow the same nitrate consumption strategy? * ***Required*** *

Marca solo un óvalo.

- Yes
- No

17. Could you give more details?

____
____
____
____
____

18. Have any players experienced any of the following adverse effects from the use of nitrates? (select more than one if necessary) * ***Required*** *

Selecciona todas las opciones que correspondan.

- [ ] None
- [ ] Pink feces
- [ ] Pink urine
- [ ] Hypotension
- [ ] Bad taste
- [ ] Gastrointestinal discomfort
- [ ] Nausea
- [ ] Vomiting
- [ ] Otros: ______________________

19. How often? * ***Required*** *

Selecciona todas las opciones que correspondan.

- [ ] Never
- [ ] Extremely rarely
- [ ] Occasionally
- [ ] Frequently

20. Are nitrates consumed in combination with other supplements or sports foods? *
* **Required**

Selecciona todas las opciones que correspondan.

- [ ] Yes
- [ ] No

21. Indicates with which supplement or sports food the nitrates are taken. *
* **Required**

Selecciona todas las opciones que correspondan.

- [ ] Caffeine
- [ ] Creatine
- [ ] Beta-alanine
- [ ] Citrulline malate
- [ ] Sodium bicarbonate
- [ ] Vitamin D
- [ ] Probiotics
- [ ] Multivitamins
- [ ] Gels
- [ ] Isotonic drinks
- [ ] Energy bars
- [ ] Sports confectionery
- [ ] Electrolites
- [ ] Whey protein
- [ ] Zinc supplement
- [ ] Calcium supplement
- [ ] Iron supplement
- [ ] Otros: ____________________

22. In addition to the supplementation provided by the club, is the consumption of natural nitrates from food monitored? * **Required** *

Marca solo un óvalo.

- ( ) Yes
- ( ) No

23. Among all the ergogenic effects prescribed in the scientific literature, which one(s) are you looking for? * ***Required***

Selecciona todas las opciones que correspondan.

- [ ] To improve health
- [ ] To improve muscle strength (e.g. to improve mechanical tension in strength exercise)
- [ ] To improve endurance performance (e.g. delay fatigue during running)
- [ ] To improve performance at high altitudes
- [ ] Otros: ______________________

24. Is the use of mouthwashes in football players monitored? * ***Required*** *

Selecciona todas las opciones que correspondan.

- [ ] No
- [ ] Yes, on the use of nitrate supplementation
- [ ] Yes, daily

25. Do you perform nitrate loading on football players? * ***Required*** *

Selecciona todas las opciones que correspondan.

- [ ] No
- [ ] Yes, for 2 days
- [ ] Yes, for 3 days
- [ ] Yes, for 4 days
- [ ] Yes, for 5 days
- [ ] Yes, for 6 days
- [ ] Yes, for 7 days
- [ ] Yes, for 7 days or more

Google no creó ni aprobó este contenido.

Google Formularios
